# High‐fat, high‐sucrose, and combined high‐fat/high‐sucrose diets effects in oxidative stress and inflammation in male rats under presence or absence of obesity

**DOI:** 10.14814/phy2.15635

**Published:** 2023-04-09

**Authors:** Jéssika Butcovsky Botto Sarter Kobi, Amanda Martins Matias, Patrícia Vasconcelos Fontana Gasparini, Suellem Torezani‐Sales, Amanda Rangel Madureira, Daniel Sesana da Silva, Camila Renata Correa, Jéssica Leite Garcia, Douglas Haese, Breno Valentim Nogueira, Arícia Leone Evangelista Monteiro de Assis, Ana Paula Lima‐Leopoldo, André Soares Leopoldo

**Affiliations:** ^1^ Postgraduate Program in Nutrition and Health, Health Sciences Center Federal University of Espírito Santo Vitória Espírito Santo Brazil; ^2^ Postgraduate Program in Physiological Science, Health Sciences Center Federal University of Espírito Santo Vitória Espírito Santo Brazil; ^3^ Postgraduate Program in Physical Education, Center of Physical Education and Sports Federal University of Espírito Santo Vitória Espírito Santo Brazil; ^4^ The Medical School São Paulo State University Botucatu São Paulo Brazil; ^5^ University of Vila Velha Vila Velha Espírito Santo Brazil; ^6^ Department of Morphology, Health Sciences Center Federal University of Espírito Santo Vitória Espírito Santo Brazil; ^7^ Center of Physical Education and Sports Federal University of Espírito Santo Vitória Espírito Santo Brazil

**Keywords:** diets, inflammation, obesity, oxidative stress, rats

## Abstract

The study examines the influence of three types of hypercaloric diets on metabolic parameters, inflammatory markers, and oxidative stress in experimental model. Male Wistar rats (*n* = 40) were randomized in control (C), high‐sucrose (HS), high‐fat (HF), and high‐fat with sucrose (HFHS) for 20 weeks. Nutritional, metabolic, hormonal, and biochemical profiles, as well as histological analysis of adipose and hepatic tissues were performed. Inflammation and oxidative stress were determined. HF model caused obesity and comorbidities as glucose intolerance and arterial hypertension. In relation to hormonal and biochemical parameters, there was no significant difference between the groups. All groups showed increased deposition of fat droplets in the hepatic tissue, even though adipocyte areas were similar. Biomarkers of oxidative stress in serum and adipose tissues were similar among the groups. HF model was effective in triggering associated obesity and comorbidities in male rats, but all hypercaloric diets were unable to promote oxidative stress and inflammation.

## Graphical abstract

(1) Hypercaloric diets can be the main contributor to the current epidemic of obesity and comorbidities. (2) The effects of a combined high‐sugar and high‐fat on inflammatory process and oxidative stress are scare and not well characterized. (3) The type of nutrient or substrate combination promotes deleterious and distinct effects in the organism in these experimental models.

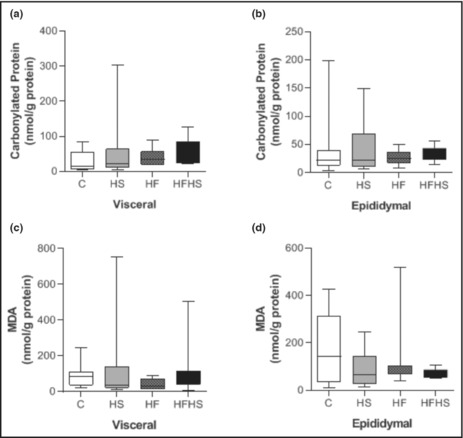

## 1 INTRODUCTION

Obesity is characterized by increased adipose tissue and is considered one of the main epidemic diseases of the 21st century (Baer et al., 2004). This multiple disease is related to several comorbidities, such as diabetes mellitus, insulin resistance, dyslipidemias, cardiovascular diseases, and certain types of cancer (Baer et al., 2004). Data from the World Health Organization (WHO) indicate that by 2016 more than 1.9 billion adults, over 18 years old, were overweight and 650 million of those were obese (WHO, 2021). Although some authors suggest that the genetic factor contributes to the development of obesity, the most studies emphasize that the prevalence of this disease occurs due to environmental factors (Rosini et al., 2012). Therefore, excessive fat accumulation results from the energy imbalance obtained from the interaction between several factors, among the diets with high energy density (Feillet‐Coudray et al., 2019).

An important alternative for studying obesity, comorbidities, and mechanisms derived from excessive body fat (BF) is the experimental obesity. Thus, obesity induced by highly palatable and energetic diets represents a reliable and appropriate model for studying the causes and consequences of human obesity. Studies have demonstrated the role of hypercaloric diets in the development of experimental models of obesity due to changes in body composition, such as increased body weight (BW) and fat deposits, changes in glycemic and lipid profiles, insulin resistance, and hypertriglyceridemia (Da Silva et al., 2010; Gamelin et al., 2016; White et al., 2013). In addition, diets characterized by the combination of high amounts of saturated fat and glucose may promote neurohormonal disorders, inflammation, and oxidative stress (Odermatt, 2011).

Oxidative stress occurs as a result of an imbalance between reactive oxygen species (ROS) formation and antioxidant defenses (Rodrigo et al., 2011). Obesity affects mitochondrial metabolism, contributing to ROS production (Serra et al., 2013). The literature has shown that hypercaloric diets results in a postprandial state of hypertriglyceridemia, hyperglycemia, and elevated levels of free fatty acids (FFAs) in the circulation, generating an oxidative stress in obesity models (Feillet‐Coudray et al., 2019; Sweazea et al., 2010).

Another important aspect is the relationship between the excess of adipose tissue and the metabolic diseases, which can lead to a chronic state of low‐grade inflammation (Herieka & Erridge, 2014). The inflammation process is a natural and essential response provided by the immune system to ensure tissue survival after tissue injury (Chandrasekharan & Sharma‐Walia, 2015). However, when inadequately or inefficiently controlled, it can become a cause of injury and disease, as in multiple sclerosis, Alzheimer's disease, rheumatoid arthritis, systemic lupus erythematosus, cardiovascular diseases, cancer, chronic respiratory disease, and diabetes (Duncan et al., 2012). Ferreira et al. (2011) investigated the effect of hypercaloric diets rich in carbohydrates and unsaturated fat on metabolic parameters and demonstrated that the nature of nutrients in a diet influences the production of proinflammatory cytokines differently in target organs and may contribute to the comorbidities due to obesity.

Thus, the key point of this study was to verify which types of hypercaloric diets (sucrose, fat, and a combination of sucrose and fat) contribute to the development of the inflammatory process and oxidative stress, as well as to investigate whether a specific nutrient or substrate combination promote deleterious and distinct effects in the organism. It was hypothesized that the hypercaloric diets would promote oxidative stress and exacerbates the inflammatory process, being this condition more evident in the hyperlipidic diet combined with high sucrose content.

## 2 MATERIALS AND METHODS

### 2.1 Animals and treatment

Experimental protocols were approved by the Research Ethics Committee of the Universidade Federal do Espírito Santo (protocol no. 08/2016). Forty male Wistar rats (110 g), 30 days old, obtained from the Animal Quarters of the Universidade Federal do Espírito Santo (Vitória, Espírito Santo, Brazil) were individually caged in rooms with regulated temperature (24 ± 2°C), humidity (55% ± 5%), and 12 h light–dark cycle. All animals had free access to water and rat chow (40 g/day). Ten animals were randomly assigned to each group: control diet (C), high‐sucrose diet (HS), high‐fat diet (HF), and high‐fat and high‐sucrose diet (HFHS). Diets were established according to Matias et al. (2018) and their composition are exposed in Table 1. The HS group had water supplemented with sucrose (300 g/L) in alternate weeks. For the calculation of the caloric intake (CI) of the HS group, the caloric energy from the water supplemented with sucrose was also quantified (1.2 kcal/mL consumed).

**TABLE 1:** Composition and nutritional values of diets.

Components (g/kg)	Diets			
	C	HS^a^	HF	HFHS
Corn	200	200	180	80
Rice	200	200	200	200
Bone meal	120	120	120	120
Sucrose	—	100	—	100
Soy oil	75	75	—	—
Lard	—	—	200	200
Gluten	200	200	200	200
Salt	3.5	3.5	3.5	3.5
Mineral mix	35	35	35	35
Vitamin mix	16.5	16.5	16.5	16.5
Inert material	150	50	45	45
Total (g)	1000	1000	1000	1000
Nutrient composition (%)				
Protein	24.8	21.8	17.8	19.2
Carbohydrate	49.6	52.3	44.6	43.4
Lipids	25.6	25.9	37.6	37.4
Energy density (kcal/g)	3.55	3.65	4.59	4.49

Abbreviations: Diets: C, normal rodent chow; HF, high‐fat; HFHS, high‐fat and high‐sucrose; HS, high‐sucrose.

^a^ Rats received diet with simple carbohydrate and water supplemented with sucrose (300 g/L) in alternate weeks. In order to calculate the caloric intake of HS, the caloric value of the sucrose diet (3.65 kcal/g) plus the caloric value of water intake with sucrose (1.2 kcal/mL).

At the end of the experimental protocol (20th week), preceding euthanasia, the animals were fasted for 12–15 h and anesthetized intraperitoneally with ketamine hydrochloride (50 mg/kg/i.p., Dopalen, Sespo Indústria and Comércio Ltda.) and xylazine hydrochloride (10 mg/kg/i.p., Anasedan, Sespo Indústria and Comércio Ltda.). Subsequently, their chests were opened by mid‐thoracotomy. The blood samples, for biochemical and hormonal measurements, were collected in Falcon® tubes, centrifuged for 10 min at 10,000 rpm (Heraeus Megafuge 16R Centrifuge, Thermo Scientific) and stored at −80°C (Coldlab Ultra Freezer CL374‐86V). The whole liver and adipose tissue fat pads were removed, weighed, frozen in liquid nitrogen, and stored at −80°C until analysis.

### 2.2 Nutritional status and body composition

To analyze whether dietary‐induced obesity was associated with alterations in nutritional behavior, food consumption (FC) was measured daily. CI was calculated weekly by the average weekly FC × dietary energetic density. Feed efficiency (FE), the ability to transform consumed calories into BW, was determined by following the formula: mean BW gain (g)/total CI (kcal). BW was recorded weekly. BF was measured based on the sum of the individual fat pad weights as follows: BF = epididymal fat + retroperitoneal fat + visceral fat. The adiposity index used to assess obesity was calculated using the following formula: adiposity index [BF/FBW] × 100.

### 2.3 Biochemical analysis

Serum glucose, total cholesterol (T‐Chol), high‐intensity cholesterol (HDL), and low‐intensity cholesterol (LDL) concentrations were measured using specific kits (Bioclin Bioquímica® and Synermed do Brasil Ltda.) and analyzed by automated biochemical equipment BS‐200 (Mindray do Brasil—Comércio and Distribuição de Equipamentos Médicos Ltda.).

### 2.4 Hemodynamics and metabolic profiles

Systemic arterial pressure (SBP) and heart rate (HR) were assessed indirectly through the tail‐cuff plethysmography method coupled with the data acquisition system (IITC INC, Life Science). The values of SBP and HR were obtained through signals of transducer coupled to the computer and analyzed in specific software (AcqKnowledge® MP100, Biopac Systems, Inc.). Three measurements were taken in each animal to obtain a mean score, and records associated with tail movement and/or other stressors that could interfere in the analysis were discarded.

Glucose intolerance was determined by the glucose tolerance test. In the 19th week of treatment, the rats underwent a 6‐h fasting period and a blood sample from the tip of the tail was collected. Basal blood glucose concentrations were measured in blood using a handheld glucometer (Accu‐Chek Go Kit—Roche Diagnostic Brazil Ltda.). Researchers administered 2 g/kg of BW of glucose solution dissolved in water to rats by intraperitoneal injection. After 30, 60, 90, and 120 min following glucose administration, blood glucose levels were measured. Glucose intolerance was evaluated by the area under the curve (AUC) for glucose. The hormones insulin and leptin were determined by enzyme‐linked immunosorbent assay (ELISA) using specific kits (Linco Research Inc.). The readings were performed on microplate reader (Asys Expert Plus Microplate Reader). The homeostatic model assessment—insulin resistance (HOMA‐IR) index was used as an index of insulin resistance and calculated according to the formula: HOMA‐IR = [glucose fasting (mmol/L) × insulin fasting (μU/mL)]/22.5.

### 2.5 Histological analysis

Histological analyses were performed on visceral adipose tissue and hepatic tissue according to the methodologies described below. The visceral adipose tissue was fixed for 24 h in 4% paraformaldehyde with 0.1 M phosphate buffer (pH 7.4). After dehydration in ethanol and clearing in xylol, the tissue was embedded in paraffin to form blocks. Sections of 5 μm were obtained using a LEICA RM2125 microtome (LEICA Biosystems Inc.) and stained with hematoxylin–eosin (H&E). Images were captured with a video camera (Evolution, Media Cybernetics, Inc.) coupled to an optical microscope (Eclipse 400, Nikon) under 40× magnification. Measurements were performed using the specific software (Image J Pro‐Plus®, Media Cybernetics). The cell area (μm^2^) was obtained, and the examiner was blinded to the experimental groups.

Livers were removed and fixed in 4% buffered formaldehyde solution for 48 h, then cross‐sectioned at 10 μm thicknesses in a −25°C cryostat (Jung CM 1860; Leica). Sections were mounted on gelatin‐coated slides and stained with Oil‐Red‐O (Sigma‐Aldrich) for detection of neutral lipids for morphometric analyses. Images were captured with a video camera (AxioCam ERc5s, Carl Zeiss) coupled to an optical microscope (AX70, Olympus Corporation) using 40× objective magnification and quantified using ImageJ software (National Institutes of Health). For each analysis, 10 different fields per animal were randomly used to calculate the average percentage of stained area (fat deposition); the examiner was blinded to the experimental groups.

### 2.6 Inflammatory profile

Interleukin‐6 (IL‐6) and adiponectin were measured in serum by ELISA using specific kits (EMD Millipore Corporation and Aldrich®), their reading was performed on Asys Expert Plus Microplate Reader (Biochrom), and expressed in pg/mL and ng/mL, respectively.

### 2.7 Biomarkers of oxidants and antioxidants

Dosages of malondialdehyde (MDA) and carbonylated protein, biomarkers of oxidative stress, were evaluated in serum and homogenate of visceral and epididymal adipose tissues.

For the quantification of MDA in the serum and homogenates of adipose tissues, 250 μL were used for 750 μL of 10% trichloroacetic acid for precipitation of proteins. Samples were centrifuged (3000 rpm; for 5 min; Eppendorf® Centrifuge 5804‐R) and the supernatant was removed. Thiobarbituric acid (TBA) was added to the samples in the ratio 1:1 and the samples were heated for 15 min at 100°C. MDA reacted with TBA in the ratio 1:2 MDA‐TBA, absorbed at 535 nm. After cooling, the reading at 535 nm was performed on Spectra Max 190 microplate reader (Molecular Devices®). The MDA concentration was obtained by the molar extinction coefficient (1.56 × 105 M^−1^ cm^−1^) and the absorbance of the samples; the result expressed in nmol/g protein.

Protein carbonylation was quantified, being 100 μL of serum and homogenates of adipose tissues to 100 μL 2,4‐dinitrophenylhydrazine (10 mM in 2 M HCl) used. The samples were incubated for 10 min at room temperature and 50 μL of NaOH (6 M) was added; subsequently, the substance was again incubated for 10 min at room temperature. The reading was performed at 450 nm on a Spectra Max 190 microplate reader (Molecular Devices®) and the result was obtained from the absorbance of samples and the molar extinction coefficient (22,000 M^−1^ cm^−1^). The results were expressed in nmol/mg protein.

The antioxidant enzyme activities of superoxide dismutase (SOD) and catalase (CAT) were evaluated in serum. SOD activities were determined, based on the inhibition of a superoxide radical reaction with pyrogallol. Changes in absorbance of the reaction solution at 420 nm were determined after 1 min. The values are expressed as units per milligram of protein per minute. CAT activities were evaluated by following the decrease in the levels of hydrogen peroxide. The absorbance values were measured at 240 nm and expressed as pmol/mL/min.

### 2.8 Statistics

The results were expressed as mean ± standard error of the mean (SEM) or median ± interquartile range, submitted to the Kolmogorov–Smirnov test to determine adherence to normality and analyzed using one‐way analysis of variance (ANOVA) followed by the Tukey's and/or Kruskal–Wallis post hoc test. The weekly evolution of BW and glucose tolerance test were submitted to two‐way ANOVA for the repeated measures and complemented by the Tukey's post hoc test. A power calculation was performed using the G*Power software (University of Düsseldorf) to determine the sample size per group. The sample size for four groups was calculated using a one‐way ANOVA test with input parameters as follows: effect size of 0.57, α error probability of 0.05, and a statistical power of 80%. Then, the total sample size required for the current study was 40 (10 rats per group). All data were analyzed by a two‐sided significance level of 5% (0.05).

## 3 RESULTS

### 3.1 Nutritional status and body composition

Regarding the nutritional profile, the FC (g) of the HS and HFHS groups were smaller than the C group (26% and 21%, respectively). In addition, the HF group had higher FC than the HS group (HF: 19.2 ± 1.0 vs. 16.1 ± 0.6, *p* < 0.05). The daily CI of the HS group was calculated based on the sum of the CI from FC (58.9 ± 2.1 kcal) and the mean CI from the sucrose water (data not shown). Thus, the CI of this group was higher than the C and HFHS groups (*p* < 0.05). Despite this, the animals in the HS group showed lower FE than the other groups (HS < C, HF and HFHS). The experimental groups HF and HFHS showed higher values of FE when compared to C (14% and 21%, respectively) and HS (31% and 39%, respectively) (Table 2).

**TABLE 2:** Nutritional status, body composition, and histological analysis of visceral adipose tissue (mean ± SEM).

Variables	Experimental groups			
	C	HS	HF	HFHS
FC (g/day)	21.9 ± 0.7	16.1 ± 0.6^a^	19.2 ± 1.0^b^	17.3 ± 0.5^a^
CI (kcal/day)	77.6 ± 2.5	92.2 ± 2.7^a^	88.0 ± 4.4	77.8 ± 2.4
FE (%)	3.85 ± 0.09	3.35 ± 0.06^a^	4.38 ± 0.11^a^ ^,^ ^b^	4.64 ± 0.11^a^
IBW (g)	107 ± 4	111 ± 4.2	113 ± 4	113 ± 4
FBW (g)	525 ± 17	543 ± 16	658 ± 43^a^ ^,^ ^b^	619 ± 27
Weight gain (g)	418 ± 16	432 ± 16	545 ± 40^a^ ^,^ ^b^	506 ± 24
Epididymal (g)	11.5 ± 0.6	10.9 ± 0.9	13.3 ± 1.1	13.7 ± 1.3
Visceral (g)	11.3 ± 0.7	10.8 ± 0.9	17.7 ± 1.7^a^ ^,^ ^b^	15.8 ± 1.8
Retroperitoneal (g)	21.5 ± 1.2	22.2 ± 2.9	40.2 ± 5.2^a^ ^,^ ^b^	33.6 ± 2.5
Body fat (g)	44.3 ± 1.9	43.9 ± 4.2	71.2 ± 7.4^a^ ^,^ ^b^	63.1 ± 5.2
AI (%)	8.44 ± 0.31	7.96 ± 0.54	10.6 ± 0.5^a^ ^,^ ^b^	10.1 ± 0.43^b^
Visceral area (μm^2^)	1288 ± 234	1358 ± 397	1562 ± 410	1171 ± 233

Abbreviations: Each group: 10; AI, adiposity index; C, control diet; CI, caloric intake; FC, food consumption; FBW, final body weight; FE, feed efficiency; HF, high‐fat diet; HFHS, high‐fat and high‐sucrose diet; HS, high‐sucrose diet; IBW, initial body weight; SEM, standard error of the mean.

^a^ When compared to the C; one‐way analysis of variance (ANOVA) for independent samples followed by Tukey's post hoc test.

^b^ When compared to the HS; one‐way analysis of variance (ANOVA) for independent samples followed by Tukey's post hoc test.

The BWs of the HF rats were significantly higher than those of the C rats in the seventh week of treatment, which remained significantly greater during the 20 weeks of the experiment. In addition, in the seventh week the HF group also had a higher BW than HS group until the end of the experimental protocol. Furthermore, the results show that HFHS developed greater BWs than C in the last weeks (17th, 19th, and 20th week) and HS in alternate weeks (13, 15, 17, 19, and 20). There was no significant difference in BW between the HS and C groups and between the HF and HFHS groups throughout the experimental protocol. At the end of the experimental protocol (20th week), rats fed with HF gained more BW (an elevation of 30% and 26%) compared to those fed with C and HS. The HF diet promoted a substantial elevation in the visceral and retroperitoneal fat pads compared to C (57% and 87%, respectively) and HS (64% and 81%, respectively) diets. Similarly, the results indicate that total fat (sum of fat deposits) was higher in HF when compared to C (61%) and HS (62%), reflecting directly a higher adiposity index. Considering also this parameter, the HFHS presented higher AI in relation to HS, but without alterations in BF (Table 2). The HFHS diet intervention did promote alterations in FBW and weight gain after the end of experiment (Week 20).

### 3.2 Hemodynamics, metabolic, and hormonal analysis

Hemodynamics, metabolic, and hormonal parameters associated with obesity are summarized in Table 3. The biochemical, hormonal, and hemodynamic parameters including glucose (*p* = 0.45), T‐Chol (*p* = 0.48), HDL (*p* = 0.63), LDL (*p* = 0.42), insulin (*p* = 0.05), HOMA‐IR (*p* = 0.07), leptin (*p* = 0.08), and HR (*p* = 0.65) were similar among the groups. The results of the glucose tolerance test showed that glycemic levels were significantly elevated in the HF (27% and 25%, respectively) and HFHS (19% and 21%, respectively) groups at 30 and 60 min after glucose administration compared to C. In addition, HF presented higher glucose after 30 min than HS (22%). The area under the glycemic curve (AUC) of the HF and HFHS groups showed higher values than C (C = 1234 ± 65, HF = 1521 ± 57, HFHS = 1460 ± 45, *p* < 0.05). HF also presented an elevation in AUC in relation to HS rats (HF = 1521 ± 57 vs. HS = 1320 ± 35, *p* < 0.05) (data not shown). Furthermore, rats fed with HS, HF, and HFHS diets for 20 weeks had an approximately 17%, 12%, and 16% higher SBP, respectively, than group C (*p* < 0.001).

**TABLE 3:** Hemodynamics, metabolic, and hormonal parameters (mean ± SEM).

Variables	Experimental groups			
	C	HS	HF	HFHS
Glucose (mg/dL)	108 ± 2	110 ± 4	115 ± 3	113 ± 3
T‐Chol (mg/dL)	81.9 ± 3.6	74.9 ± 4.6	79.3 ± 5.6	73.1 ± 3.2
HDL (mg/dL)	27.1 ± 1.4	24.6 ± 0.9	27.6 ± 3.1	25.4 ± 1.4
LDL (mg/dL)	9.78 ± 0.78	10.9 ± 1.3	10.7 ± 1.3	8.61 ± 0.53
Insulin (ng/mL)	1.86 ± 0.13	1.77 ± 0.15	2.19 ± 0.16	2.39 ± 0.23
HOMA‐IR	19.9 ± 1.6	19.7 ± 2.1	24.8 ± 1.9	26.4 ± 2.6
Leptin (ng/mL)	12.1 ± 2.7	12.7 ± 4.1	24.1 ± 2.8	18.9 ± 4.7
SBP (mmHg)	147 ± 3	172 ± 5^a^	164 ± 3^a^	170 ± 2^a^
HR (bpm)	426 ± 17.4	425 ± 14.1	427 ± 17.0	404 ± 13.0

*Note*: *N*: number of animals; Control diet (C; *n* = 10), high‐sucrose (HS; *n* = 10), high‐fat diet (HF; *n* = 9) or high‐fat and sucrose diet (HFHS; *n* = 9). Insulin and HOMA‐IR: C (*n* = 6); HS (*n* = 7); HF (*n* = 7) and HFHS (*n* = 7). Leptin: C (*n* = 6); HS (*n* = 5), HF (*n* = 5); HFHS (*n* = 4); SBP: (*n* = 6); HR: (9).

Abbreviations: HDL, high‐density lipoprotein; HR, heart rate; LDL, low‐density lipoprotein; SBP, systolic blood pressure; SEM, standard error of the mean; T‐Chol, total cholesterol.

^a^ *p* < 0.05 when compared to the C; one‐way analysis of variance (ANOVA) for independent samples followed by Tukey's post hoc test.

### 3.3 Histological analysis

**FIGURE 1:**
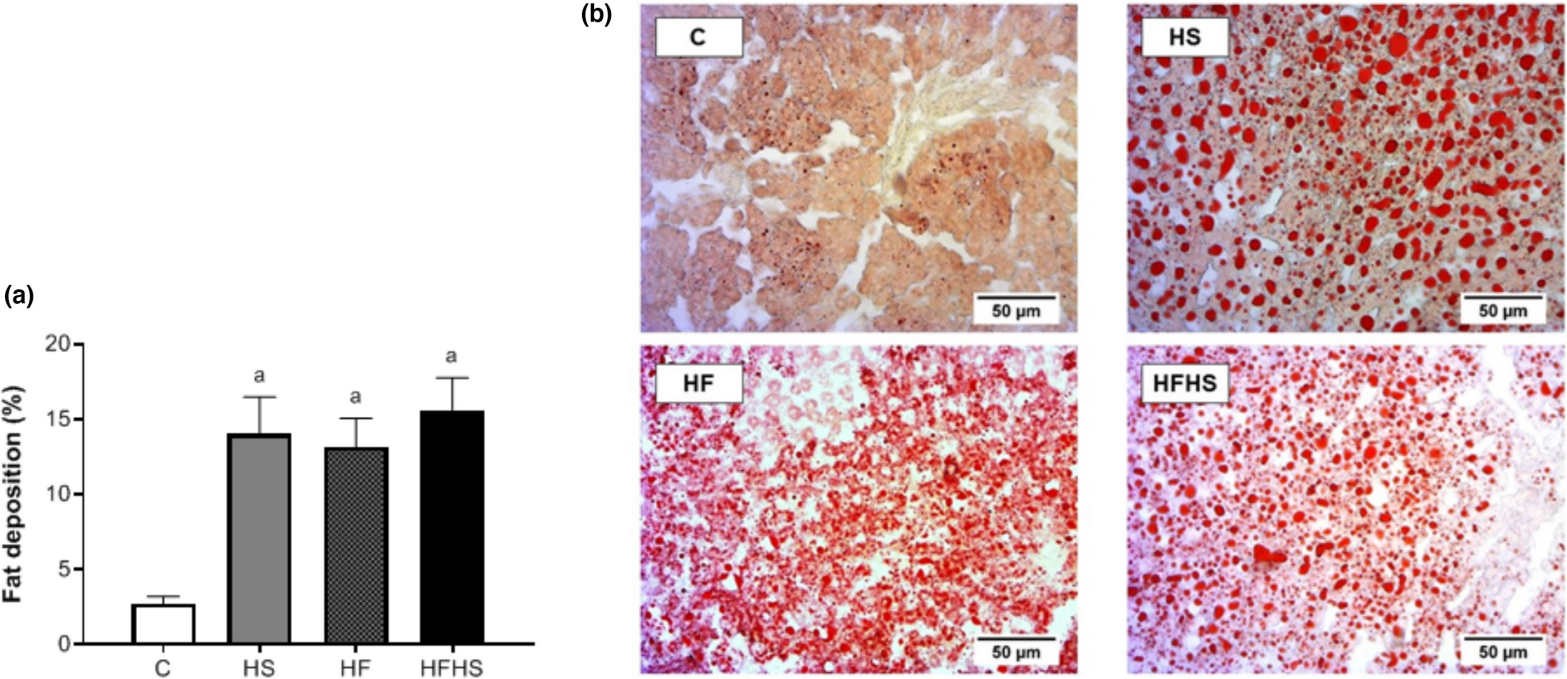
Effect of the intake of different hypercaloric diets on the induction of non‐alcoholic fatty liver disease of Wistar rats after 20 weeks. a) Representative histological images of hepatic tissue stained with oil red. Scale bar: 50 μm. b) *n* = number of animals; control (C; *n* = 7), high‐sucrose (HS; *n* = 7), high‐fat diet (HF; *n* = 7), or high‐fat and sucrose diet (HFHS; *n* = 5). Values are expressed as mean ± standard error of the mean (SEM). One‐way analysis of variance (ANOVA) for independent samples, complemented with Tukey's post hoc test. *p* < 0.05; a vs. C.

**FIGURE 2:**
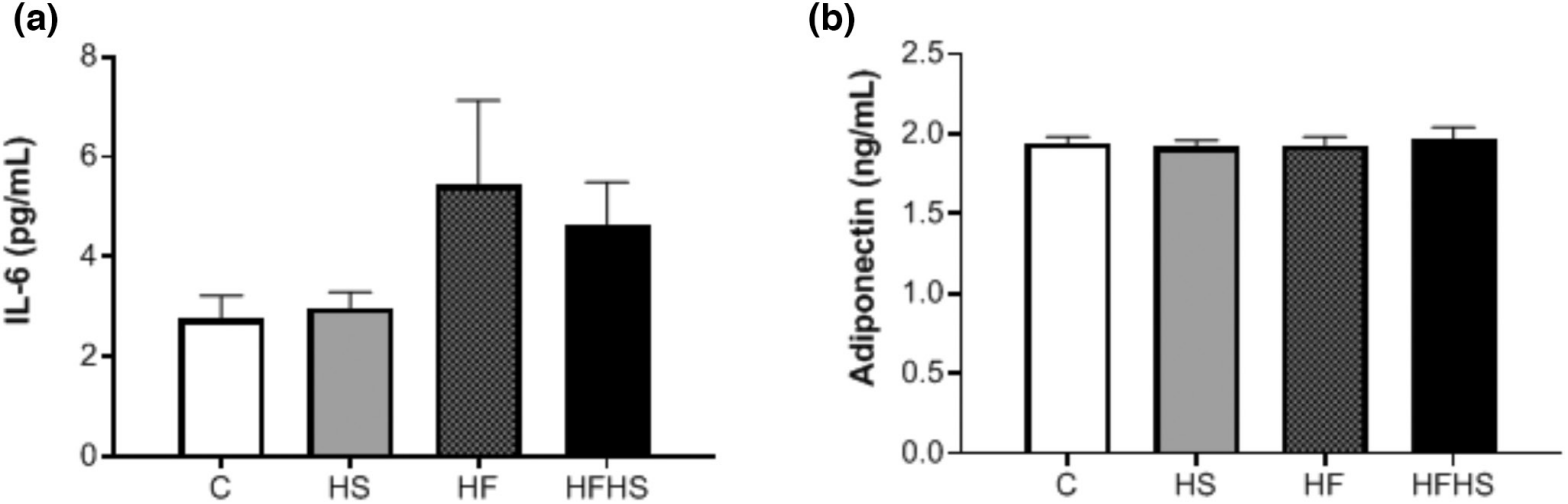
Inflammatory profile. a) Interleukin‐6 (IL‐6) and b) adiponectin. *n* = number of animals; control (C; *n* = 5), high‐sucrose (HS; *n* = 6), high‐fat diet (HF; *n* = 5), or high‐fat and sucrose diet (HFHS; *n* = 6). C (*n* = 8); HS (*n* = 10); HF (*n* = 9); HFHS (*n* = 8). Values are expressed as mean ± standard error of the mean (SEM). One‐way analysis of variance (ANOVA) for independent samples, complemented with Tukey's post hoc test.

Figures 1 and 2 present the effect of the intake of different hypercaloric diets on hepatic tissue of Wistar rats after 20 weeks. The experimental groups presented increased deposition of fat droplets in hepatic tissue when compared to group C (HS = 14.02 ± 2.48, HF = 13.17 ± 1.89, and HFHS = 15.63 ± 2.13 vs. C = 2.73 ± 0.47; *p* < 0.001) (Figure 1). Regarding visceral adipose tissue, the results showed that adipocyte areas were similar between experimental groups (*p* = 0.31; Table 2).

### 3.4 Inflammatory profile

Regarding the inflammatory profile, the results showed that there was no significant difference between the experimental groups for the serum IL‐6 (*p* = 0.16) and adiponectin (*p* = 0.93) values (Figure 2).

### 3.5 Oxidant and antioxidant biomarkers

**FIGURE 3:**
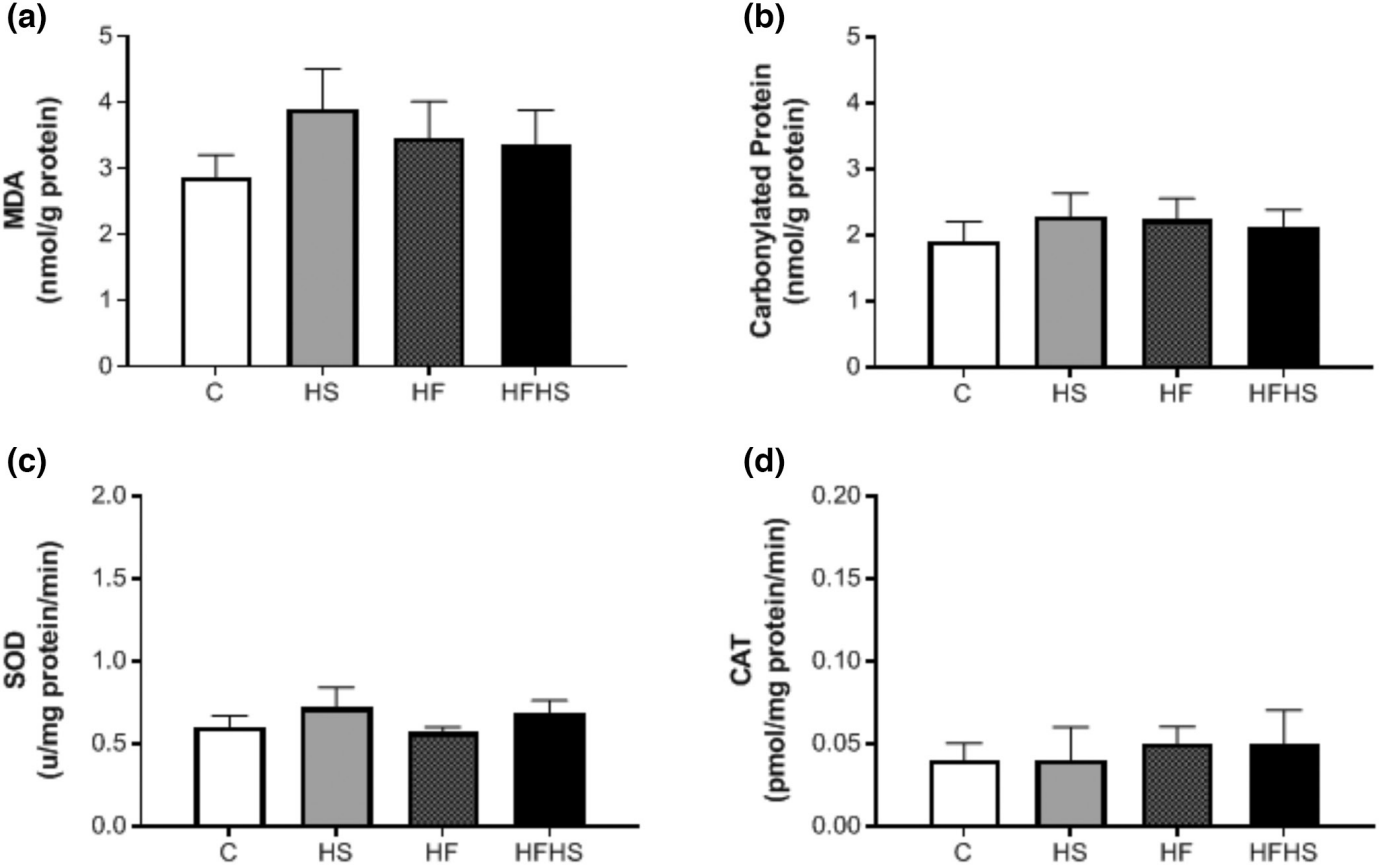
Serum biomarkers of oxidative stress and enzyme activity antioxidants. *n* = number of animals; control (C; *n* = 7), high‐sucrose (HS; *n* = 10), high‐fat diet (HF; *n* = 7), or high‐fat and sucrose diet (HFHS; *n* = 9). (a) MDA, malondialdehyde; (b) carbonylated proteins; (c) SOD, superoxide dismutase; (d) CAT, catalase. Values are expressed as mean ± standard error of the mean (SEM). One‐way analysis of variance (ANOVA) for independent samples, complemented with Tukey's post hoc test.

**FIGURE 4:**
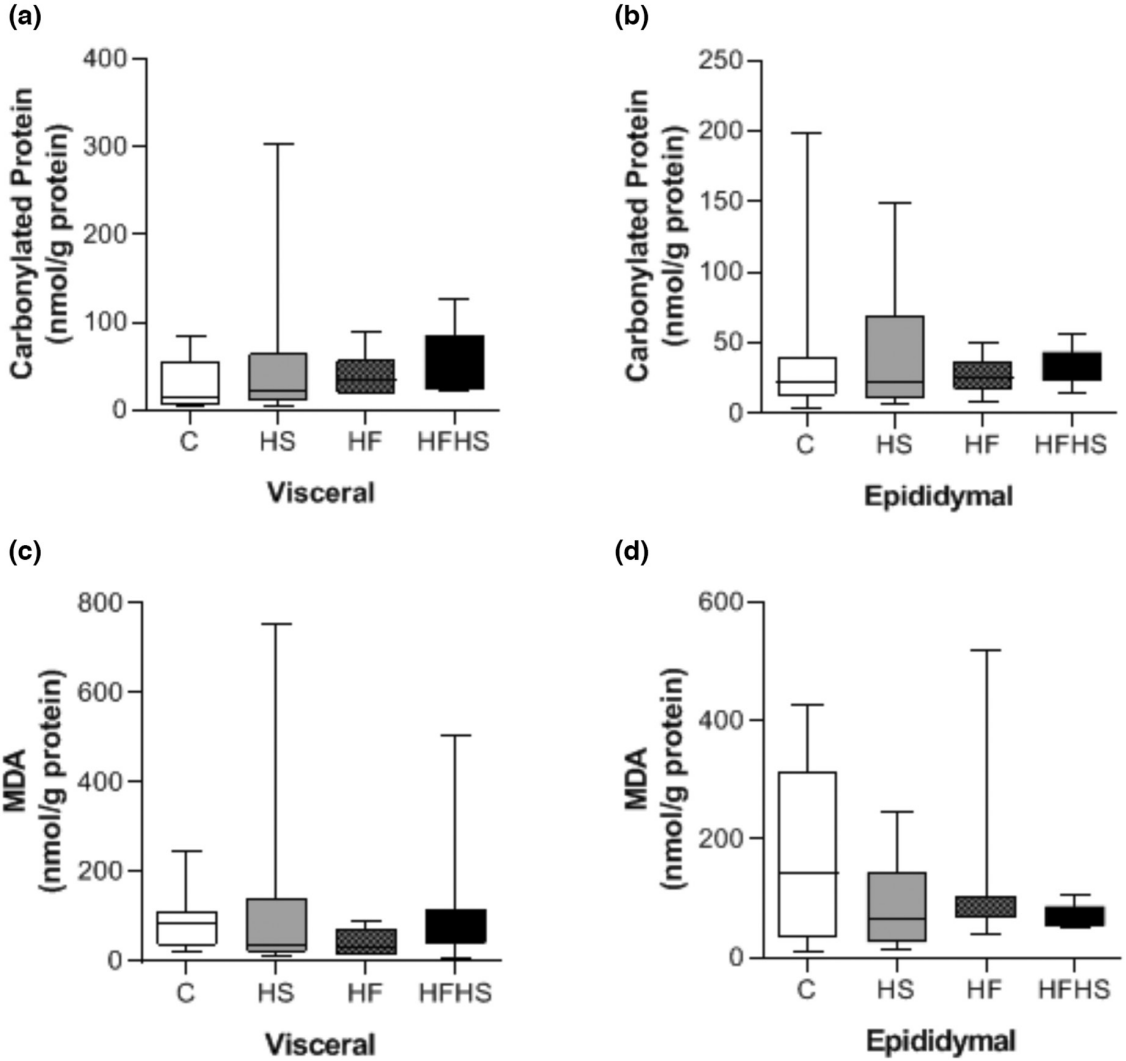
Adipose tissue biomarkers of oxidative stress and enzyme activity antioxidants. (a) Carbonylated proteins in the visceral adipose tissue. (b) MDA in the visceral adipose tissue. (c) Carbonylated proteins in epididymal adipose tissue. (d) MDA in epididymal adipose tissue. *n* = number of animals; control (C; *n* = 7), high‐sucrose (HS; *n* = 10), high‐fat diet (HF; *n* = 6), or high‐fat and sucrose diet (HFHS; *n* = 9). C (*n* = 7); HS (*n* = 8); HF (*n* = 7); HFHS (*n* = 8). Values are expressed as median ± interquartile range. Non‐parametric analysis of variance (ANOVA) for independent samples (Kruskal–Wallis).

Similarly, considering the concentrations of oxidative stress biomarkers (MDA and carbonylated proteins) and the activities of antioxidant enzymes (SOD and CAT) in serum, the results showed that there was no significant difference between the experimental groups (Figure 3a–d). No significant differences were observed between the groups for concentrations of MDA and carbonylated proteins in visceral and epididymal fat pads (Figure 4a,b).

## 4 DISCUSSION

The objective of this study was to evaluate the influence of three different types of hypercaloric diets with high amounts of sucrose and/or animal fat on metabolic, inflammatory, and oxidative stress parameters in rats. The main findings indicate that the experimental model for HF was effective in triggering obesity, glucose intolerance, and hypertension. In addition, the experimental models from diets rich in sucrose and/or combined sucrose and fat diets resulted in glucose intolerance (only the last model cited) and arterial hypertension without changes in adiposity. However, no changes were observed in the three experimental models that evidenced the process of inflammation and oxidative stress.

Experimental models of obesity by hyperlipidic diet have been extensively used in the literature to represent the etiological profile of obesity developed in humans (Da Silva et al., 2010; Gamelin et al., 2016). In our study, HF model showed a significant increase in BW in relation to the animals fed with a standard diet, remaining obese from the seventh week of treatment until the end of the experimental period (20th week). In percentage terms, the adiposity index was elevated by 20% in the HF model in relation to C. Similarly, visceral and retroperitoneal fat deposits were, respectively, 57% and 87% higher, respectively, when compared to C. Thus, the observed BW gain reflected the increase in adipose tissue. Consistent with previous investigations, hyperlipidic diets that used lard as a fat source, showed an increase in adiposity of 30%–45% (Briaud et al., 2002; Woods et al., 2003).

The rapid increase in the prevalence of obesity can be attributed in part to the availability of hypercaloric palatable foods, which result in excessive CI over need. In this way, most of the calories ingested in foods that are not used by the body are stored in adipose tissue as a source of energy reserves. Some authors report that energy from fat has a greater effect on BW gain when compared to non‐fat sources of energy (Dourmashkin et al., 2005). High lipid concentrations in the diet promoted hypertrophy and hyperplasia of the adipocyte, triggering the lipogenesis process and contributing to an increase in fat accumulation capacity (González‐Muniesa et al., 2017). It should be noted that the experimental model HL triggered obesity without entropy of adipocyte hypertrophy. Although not analyzed in this study, the results suggest that adipocyte hyperplasia may have occurred. Another possible explanation for the elevation of BW and adiposity observed in the hyperlipidic experimental model refers to the CI and food efficiency (Dourmashkin et al., 2005; White et al., 2013). We verified that the animals that received the hyperlipidic diet presented greater alimentary efficiency in comparison to C. Similar results were observed by several authors (Townsend et al., 2008; White et al., 2013), who verified higher FE in animals fed a saturated hyperlipidic diet.

The type of fat used in the composition of the diet can also contribute to greater adiposity. The predominance of saturated fatty acids gives the hyperlipidic diet a greater obesogenic effect, since this type of fatty acid triggers less direct energy generation, remaining acylated in triglycerides (TGs), and stored in adipose tissue (Buettner et al., 2007). According to Buettner et al. (2007), saturated and monounsaturated fats are able to promote more pronounced obesity and insulin resistance when compared to polyunsaturated fat. These authors argue that lard (a mixture of saturated and monounsaturated fat), a type of fat used for the composition of the hyperlipidic diet in our study, is the most recommended for the generation of a valid obese rodent model with metabolic alterations. In agreement, our study used the same type of fat for the composition of the hyperlipidic diets and evidenced greater deposition of fat droplets in the hepatic tissue of the hyperlipidic experimental models when compared to the control.

Recent study shows that excessive fat associated with sugar promotes elevated BW gain (Martire et al., 2015), but in disagreement with the initial hypothesis of our study, HFHS showed elevation of BW only in the final weeks (17th, 19th, and 20th week). This dietary intervention, however, resulted in an increase on visceral and retroperitoneal fat deposits, as well as in the adiposity index. This finding corroborates studies that have verified an increase in the prevalence of metabolically obese individuals, who present metabolic disorders similar to obese people, however, with a weight profile similar to eutrophic individuals (Cao et al., 2012).

The literature is controversial regarding experimental obesity models based on a high sugar diet. In our study, the HS model did not lead to a gain in BW and an increase in adiposity compared to C. In contrast, Malafaia et al. (2013) observed elevation in BW of animals receiving a 30% sucrose diet from the second week of experimental protocol. However, Kanazawa et al. (2003) examined the effects of a sucrose diet (60% of dietary calories) on BW gain and after 2 weeks, they found no higher BW in this group when compared to the group that received standard diet. Cao et al. (2012) have showed that Sprague Dawley rats fed with 35% dietary calories from sugar for 20 weeks had similar BW. The lower FE presented by this model may explain the absence of a significant increase in BW and fat deposits.

Obesity is a chronic metabolic disease that promotes metabolic disorders such as glucose intolerance, hyperinsulinemia, insulin resistance, hyperleptinemia, dyslipidemia, hepatic steatosis, and hypertension (Malafaia et al., 2013). In our study, the AUC of the hyperlipidic models (HF and HFHS) was significantly higher in relation to group C, suggesting that these experimental models triggered glucose intolerance. The mechanisms by which a diet high in saturated fat and sugars, as well as fat combined with sugar, induce changes in the glucose profile, may be related to a reduction in the number of insulin receptors and the activity of the glucose transport system, as well as to the intercellular metabolism of glucose (Olefsky & Saekow, 1978). In addition, despite the findings of our study showing no changes in the serum values of T‐Chol, HDL and LDL, all dietary interventions promoted greater deposition of fat droplets in the hepatic tissue due to the elevation of FFAs in the bloodstream (Goldberg & Ginsberg, 2006), which caused a clinical condition called non‐alcoholic fatty liver disease (Feillet‐Coudray et al., 2019). Another important aspect, as previously reported, was an indicator of arterial hypertension. In this sense, although only the HF model has triggered obesity, our results indicate that the three proposed experimental models led to elevation of blood pressure levels, demonstrating that the intake of hypercaloric diets is a risk factor for the development of arterial hypertension, regardless of obesity (Matias et al., 2018). When fat mass increases, insufficient irrigation can lead to lack of oxygen and to cell necrosis. The process of phagocytosis to eliminate these dead cells results in increased inflammatory infiltration. Thus, inflammation can occur when the energy supply begins to exceed the storage capacity of adipocytes and, as a result, the hypertrophy caused leads to a higher release of adipokines as proinflammatory cytokines such as IL‐6 in low‐grade chronic inflammation (Cotillard et al., 2014). However, in our study, there was no change in serum IL‐6 and adiponectin levels that characterized inflammation and a possible explanation can be due to the absence of adipocyte hypertrophy in these models.

The literature shows that the ingestion of hypercaloric diets with high fat and/or carbohydrate content is a contributing factor in the generation of ROS during the pathogenesis of obesity and associated risk factors, evidencing the relationship between increased energy intake and the capacity to induce oxidative stress (Arias‐Chávez et al., 2022; Feillet‐Coudray et al., 2019; Renaud et al., 2014). Lushchak and Storey (2021) have classified the oxidative stress as low intensity, intermediate intensity, and strong intensity oxidative stress, which depends on dose; in our case, could be the type of energetic substrate and the duration of experimental protocol. In addition, the authors state that zones of low intensity and transition to intermediate intensity oxidative stress can occur both an increase in ROS‐inducible ROS‐sensitive parameters like the activity of antioxidant enzymes and increase in the levels of ROS‐modified substances such as lipid peroxides. Therefore, mild oxidative stress, but not strong stress, typically leads to upregulation of antioxidant systems to increase protection of organisms from the damaging effects of ROS. This appears to be the case in our results, since as provided that the dietary interventions were not able to induce oxidative stress, because we did not observe pronounced metabolic, hormonal changes, and elevation of oxidative biomarkers. In this context, the excess of energy substrates from different types of diet is associated with oxidative damage to macromolecules such as lipids (lipid peroxidation or lipid peroxidation) and proteins (carbonylation and/or nitration) because they increase the production of ROS, which alter the chemical structure and/or biological function, inducing cell death and consequent deleterious effects (Fernández‐Sánchez et al., 2011).

Bayliak et al. (2022) have observed that male mice fed high‐fat high‐fructose diet (45% kcal fat, 15% kcal fructose) presented mild oxidative stress with higher activities of primary antioxidant enzymes namely CAT, glutathione peroxidase (GPx), and glutathione‐*S*‐transferase. In disagreement with the literature, our findings showed no significant differences between the experimental groups for the levels of MDA and carbonylated proteins in serum and adipose tissue, as well as, the activities of antioxidant enzymes (SOD and CAT) did not present alterations, demonstrating that dietary interventions did not lead to oxidative stress.

The absence of oxidative damage can be attributed to the adipocyte area in our experimental models, which was similar among dietary interventions. Adipocyte hypertrophy is associated with reduced blood flow with consequent hypoxia, infiltration, and activation of macrophages (ROS source) and inflammatory cytokines in adipose tissue, such as tumor necrosis factor alpha (TNF‐α) and IL‐6, which increase the activities of subunit constituents of the enzyme NADPH oxidase and the production of superoxide anion (Tilg & Moschen, 2006). In addition, the absence of hyperglycemia and hyperleptinemia may also explain the similar levels of oxidative stress biomarkers. The condition of insulin resistance, hyperglycemia, and the consequent overload of intracellular glucose increase via glycolysis, as well as the proton gradient across the inner mitochondrial membrane, leading to an escape of electrons with superoxide anion formation (Choi et al., 2008). Hyperleptinemia, in turn, is responsible for the activation of macrophages, production of TNF‐α and NO synthase, in addition to the proliferation and migration of endothelial cells, resulting in increased superoxide generation (Izadi et al., 2022; Mangge et al., 2017). Izadi et al. (2022) have shown that high‐fat high‐fructose diet promotes elevation of plasma leptin level and the hypothalamic leptin content, oxidative stress marker (through the MDA level increment), indicating that the rise of pro‐inflammatory metabolites is induced through the high‐fat high‐fructose consumption and stimulates leptin secretion. The increased leptin level stimulates ROS production and induces oxidative stress (Berger & Polotsky, 2018).

Nevertheless, studies suggest that the amount and types of lipids in the diet affect the sensitivity of the cells to lipid peroxidation and, consequently, to oxidative damage (Thomas & Rudel, 1996). Thus, there is evidence that whereas polyunsaturated fatty acids can undergo oxidation and result in products that can be toxic to cells (Halliwell & Chirico, 1993), the saturated fatty acids, used in the formulation of our hyperlipidic diets, demonstrate less susceptibility to oxidation than unsaturated fatty acids (Varghese & Oommen, 2000).

Antioxidants have high oxidative stability due to their molecular structure and, therefore, play a fundamental role, inhibiting or reducing the oxidative reaction and damages caused by its deleterious action (Broinizi et al., 2008). Thus, antioxidant enzymes such as reduced glutathione (GSH), glutathione peroxidase (GSH‐Px), glutathione reductase (GSH‐Rd), SOD, and CAT constitute a supportive defense against ROS (Miki et al., 2018). At first, we expected that the serum activity values of SOD and CAT enzymes were high in the attempt of the organism to combat a possible exacerbated production of ROS by the influence of hypercaloric diets, explaining the absence of changes in the stress markers oxidative. However, the serum activity of these enzymes was similar among the experimental groups, demonstrating that antioxidant defenses remain unchanged on the influence of dietary interventions. In addition, the current study did not show comorbidities. The literature has emphasized that the imbalance between ROS production and antioxidant agents has been strongly correlated with the development of different cardiovascular pathologies (Zalba & Moreno, 2022).

In summary, the present study demonstrates that hyperlipidic model was effective in triggering obesity and associated comorbidities, but all experimental models of hypercaloric diets were unable to promote oxidative stress and inflammation.

### 4.1 Study limitations

The use of only male rats can be considered weakness of the current study, because it is essential to study and/or to compare the subject of the study “Effect of Hypercaloric Diets on Oxidative Stress and Inflammation” in different experimental protocols regardless of sex, that is, sex should not be considered a factor that could bias the results.

## AUTHOR CONTRIBUTIONS

Conception and design, data acquisition, analysis and/or interpretation: Jéssika Butcovsky Botto Sarter Kobi, Amanda Martins Matias, Patrícia Vasconcelos Fontana Gasparini, Suellem Torezani‐Sales, Amanda Rangel Madureira, Daniel Sesana da Silva, Camila Renata Correa, Jéssica Leite Garcia, Douglas Haese, Breno Valentim Nogueira, Arícia Leone Evangelista Monteiro de Assis, Ana Paula Lima‐Leopoldo, and André Soares Leopoldo; Significant participation in drafting the article or revising it critically for important intellectual content: Jéssika Butcovsky Botto Sarter Kobi, Amanda Martins Matias, Patrícia Vasconcelos Fontana Gasparini, Suellem Torezani‐Sales, Amanda Rangel Madureira, Daniel Sesana da Silva, Camila Renata Correa, Jéssica Leite Garcia, Douglas Haese, Breno Valentim Nogueira, Arícia Leone Evangelista Monteiro de Assis, Ana Paula Lima‐Leopoldo, and André Soares Leopoldo; Funding Acquisition: André Soares Leopoldo; Supervision: Ana Paula Lima‐Leopoldo and André Soares Leopoldo; Final approval of the version to be published: Jéssika Butcovsky Botto Sarter Kobi, Amanda Martins Matias, Patrícia Vasconcelos Fontana Gasparini, Suellem Torezani‐Sales, Amanda Rangel Madureira,Daniel Sesana da Silva, Camila Renata Correa, Jéssica Leite Garcia, Douglas Haese, Breno Valentim Nogueira, Arícia Leone Evangelista Monteiro de Assis, Ana Paula Lima‐Leopoldo, and André Soares Leopoldo; Agreement to be accountable for all aspects of the work in ensuring that questions related to the accuracy or integrity of any part of the work are appropriately investigated and resolved: Jéssika Butcovsky Botto Sarter Kobi, Amanda Martins Matias, Patrícia Vasconcelos Fontana Gasparini, Suellem Torezani‐Sales, Suellem Torezani‐Sales, Amanda Rangel Madureira, Daniel Sesana da Silva, Camila Renata Correa, Jéssica Leite Garcia, Douglas Haese, Breno Valentim Nogueira, Arícia Leone Evangelista Monteiro de Assis, Ana Paula Lima‐Leopoldo, and André Soares Leopoldo.

## CONFLICT OF INTEREST STATEMENT

The authors declare that they have no competing interests.

## ETHICS STATEMENT

Experimental protocols were approved by the Research Ethics Committee of the Universidade Federal do Espírito Santo (protocol no. 08/2016).
